# Exploring the Brassinosteroid Signaling in Monocots Reveals Novel Components of the Pathway and Implications for Plant Breeding

**DOI:** 10.3390/ijms21010354

**Published:** 2020-01-05

**Authors:** Damian Gruszka

**Affiliations:** Institute of Biology, Biotechnology and Environmental Protection, Faculty of Natural Sciences, University of Silesia, Jagiellonska 28, 40-032 Katowice, Poland; damian.gruszka@us.edu.pl; Tel.: +48-32-2009-482

**Keywords:** brassinosteroid, breeding, cereals, crops, crosstalk, monocots, signaling

## Abstract

Brassinosteroids (BRs) are a class of steroidal phytohormones which are key regulators of diverse processes during whole life cycle of plants. Studies conducted in the dicot model species *Arabidopsis thaliana* have allowed identification and characterization of various components of the BR signaling. It is currently known that the BR signaling is interconnected at various stages with other phytohormonal and stress signaling pathways. It enables a rapid and efficient adaptation of plant metabolism to constantly changing environmental conditions. However, our knowledge about mechanism of the BR signaling in the monocot species is rather limited. Thus, identification of new components of the BR signaling in monocots, including cereals, is an ongoing process and has already led to identification of some monocot-specific components of the BR signaling. It is of great importance as disturbances in the BR signaling influence architecture of mutant plants, and as a consequence, the reaction to environmental conditions. Currently, the modulation of the BR signaling is considered as a target to enhance yield and stress tolerance in cereals, which is of particular importance in the face of global climate change.

## 1. Introduction

In the modern agricultural practices crops are grown at high planting density and with relatively high nitrogen supply. These conditions promote stem elongation which may lead to plant lodging under unfavorable weather conditions. Therefore, a search for semi-dwarf cultivars of cereal crops is needed to avoid plant lodging and ensure high yields, especially in the face of ongoing climate change [1,2]. Indeed, the semi-dwarf, erect phenotype of cereal crops was reported to improve grain yield under the high-density planting conditions, even without extra fertilizer supply, as it enables more efficient light capture [3,4,5]. Thus, the increase in the planting density is considered as a major solution of the global need for enhancement in crop yield on limited arable lands to feed the global population [6]. Leaf angle is one of the key parameters determining cereal crop architecture and yield [7]. Thus, breeding cereals for more erect leaves is a strategy for the crop productivity improvement [8]. Brassinosteroids (BRs) play a prominent role in hormonal crosstalk which regulates this parameter in cereals [7,9,10,11]. In the past, application of semi-dwarf cultivars of rice (*Oryza sativa*) and wheat (*Triticum aestivum*) which were related mostly with the gibberellin metabolism and the BR signaling-related barley (*Hordeum vulgare*) mutant ‘uzu’ has significantly contributed to the yield increase, known as the ‘Green Revolution’ [12,13,14]. 

However, the high yielding cereal crop varieties were developed mainly for optimal environmental conditions of plant cultivation. Taking into account the current climate changes, priority in the modern breeding programs should be given to developing and breeding the stress-tolerant crop cultivars [15,16]. Importantly, reports published during the last few years presented some promising results which indicated that mutants defective in the BR metabolism from the monocot species *Brachypodium distachyon* (purple false brome), rice and barley show improved tolerance to drought [17,18,19,20]. These results are particularly important given the fact that drought belongs to the most limiting abiotic conditions for crop yield [15,21,22]. Therefore, currently the BR signaling is considered as an ideal target pathway for biotechnological modification to improve crop yield and stress tolerance, especially in the face of global climate change [23,24,25]. 

As a result of intensive research conducted for the last three decades in the model, dicot species *Arabidopsis thaliana* the BR signaling is one of the best described molecular processes in plants [25,26,27,28,29,30]. The intensive studies conducted with various approaches in Arabidopsis led to identification of numerous components of the BR signaling [26,31,32]. Moreover, recent reports indicated that various components of the BR signaling function as nodes of interactions with diverse signal transduction pathways of other phytohormones or environmental cues [25,33,34]. These integration hubs allow for a coordinated regulation of diverse physiological processes in reaction to environmental and stress conditions, as well as a rapid and efficient plant adaptation [25,29]. However, the network of the signaling interactions (crosstalk) appears to be very complicated, and therefore remains not fully understood. 

When compared with the BR signaling model described in Arabidopsis, our current knowledge about mechanism of the BR signaling in monocots (including cereal crops) is still rather limited [24,35,36]. Among the monocots, the BR signaling process has been described to the greatest degree in rice [25] and will be presented as a model and reference for other monocots in this review (an updated model of the BR signaling and the BR-dependent regulation of gene expression in rice will be presented in the consecutive figures). However, even in rice a detailed mechanism of the BR signaling, especially the BR-induced protein phosphorylation/dephosphorylation-mediated relay which occurs in the cytoplasm, remains not fully understood [37,38]. The rice components of the BR signaling were characterized either by ‘forward’ or ‘reverse-genetics’ approaches [21,24]. Several components of the BR signaling pathway in rice, such as OsBRI1 (Brassinosteroid-Insensitive1), OsBAK1 (BRI1-Associated receptor Kinase1) which form a core of the transmembrane BR receptor complex [3,39,40,41], OsGSK1 and OsGSK2 (Glycogen Synthase Kinases) which play a role of the major negative regulators of the BR signaling [42], and the OsBZR1 (Brassinazole-Resistant1) transcription factor which plays a pivotal function in the BR-dependent regulation of gene expression [43] were described as orthologs of Arabidopsis counterparts and as playing conserved functions [24]. However, orthologs of the BR signaling components in Arabidopsis, such as the PP2A (Protein Phosphatase 2A) and BSU (BRI1-Supressor1) phosphatases, have not been identified in rice yet [24,38,44]. Interestingly, several components of the BR signaling in rice, such as OsLIC (Leaf and Tiller Angle Increased Controller), OsDLT (DWARF and Low-Tillering), ELT1 (Enhanced Leaf inclination and Tiller number1), OsTUD1 (Taihu Dwarf1) U-box E3 ubiquitin ligase (also known as Erect Leaf1, ELF1), OsRAVL1 (Related to ABI3/VP1 RAV-Like1), OsGW5 (Grain Width5), and OsPRA2, which all will be described in this review, do not have orthologs in Arabidopsis [21,25,45,46,47,48,49,50,51]. This intriguing result indicates that some parts of the BR signaling pathway and the BR functions are specific for rice and other monocots [24].

It is known that BRs control architecture of plants (including cereals) and regulate the source-sink relationship to influence yield. Moreover, BRs play an important role in regulation of grain yield and other agronomic traits which are related to yield [23,52]. Thus, a fine-tuning of the BR signaling or biosynthesis may be a practical strategy for developing the high-yielding cereal germplasm [24]. Hence, identification and mutational analysis of the BR signaling-related genes in cereals remains very important, and the mutation-based modulation of the BR-related plant phenotype is still required for an efficient breeding of cereals [25]. The identification and mutational analysis of the BR signaling components in cereals may be beneficial because of at least two reasons: it may allow isolation of mutants with semi-dwarf and erect phenotype which is highly desired in the modern breeding programs due to the potential for increased planting density and improved drought tolerance. Secondly, research on the BR signaling in rice and other cereals has already allowed novel components of the pathway and new functions of these components to be revealed. These results broadened the view of the BR signaling which was obtained in the model species Arabidopsis. 

## 2. BR Signaling Initiation and Its Regulation in Monocots

**Figure 1:**
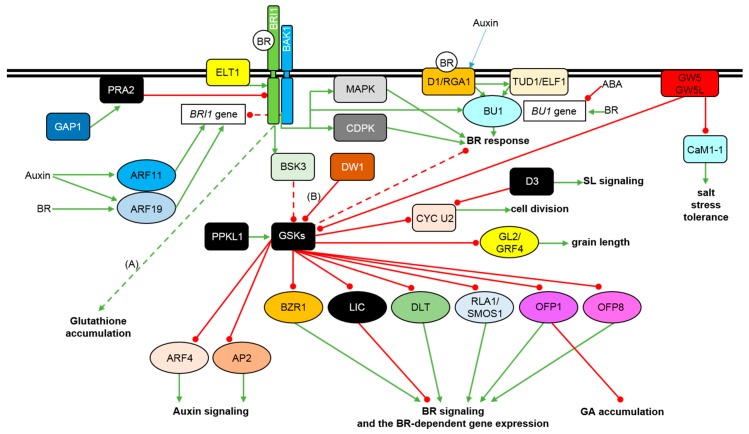
Model of the BR perception, signaling initiation, and regulation of the signal transduction in rice and other monocots. The BR molecule is depicted as the white circle with the ‘BR’ letters in it. Double black line represents the plasma membrane. Transcription factors are shown as ovals, other proteins are depicted as rectangles. Green arrows indicate stimulation, whereas red lines with the bullet points represent suppression. Detailed description is given in the text. Some of the BR responses and interactions were described in barley (**A**) and sorghum (**B**).

The BR signaling in rice is initiated by perception of the BR molecule by the OsBRI1 receptor kinase [39,40]. It was reported that transcription of the *OsBRI1* gene is repressed by the exogenous BR application in a feedback manner [39] (Figure 1). 

A series of rice mutants (named *d61*) were reported to carry mutations in the *OsBRI1* gene. The *d61* mutants represent different degree of growth reduction and erect phenotype, and loss-of-function mutations cause semi-dwarfism, erect stature, the BR insensitivity, and in some cases sterility [3,39,40,47]. Ten different alleles of the *OsBRI1* gene have been identified and characterized, and the mutations cause changes in various domains of the OsBRI1 receptor [47]. Interestingly, even in the *d61-4* mutant which carries a null allele and shows severe defects in aerial organs, root system of this genotype remains relatively normal. Based on a gene expression analysis it was reported that two homologous *OsBRI1*-like genes may play redundant roles in the BR perception in rice roots [40]. It may indicate that different organs or tissues may have different BR sensitivities or signaling pathways [53]. Importantly, although the loss-of-function mutant showed the significant reduction of plant height, a little effect on the mutant fertility was observed. This renders the *OsBRI1* gene an ideal candidate for obtaining mutant alternatives to gibberellic acid (GA)-related ones [3,54]. The *d61-7* mutant shows the mildest phenotypic effects, including semi-dwarfism and erect leaves. Interestingly, panicles of this mutant are longer and bear more grains, although the grain size is reduced. Grain yield of this mutant increased proportionally with the planting density. At high planting density an average grain yield of this mutant was comparable with the wild-type cultivar, but surprisingly the mutant produced more above-ground biomass per area [47]. The *OsBRI1-*knockdown lines showed erect leaves, but height of the plants, morphology of panicles and grains were normal. Importantly, grain yield of the rice lines was ca. 30% higher than that of the reference cultivar at high planting density [3]. As far as the hormonal crosstalk is concerned, BR sensitivity in rice may be improved by auxin. It is known that auxin can induce the *OsBRI1* gene transcription through direct binding of its promoter by the OsARF11 and OsARF19 transcription factors. Importantly, expression of the *OsARF11* gene is induced by auxin, and the *OsARF19* gene is strongly enhanced by auxin and BR [55,56,57]. Thus, the OsARF11 and OsARF19 transcription factors regulate plant height and leaf angle in rice and constitute points of crosstalk between the auxin and BR signaling pathways [57] (Figure 1). Recently, structural and functional analysis of the *TaBRI1* gene and encoded protein in wheat (*Triticum aestivum*) has been performed. In the experiments an interaction of the TaBRI1 protein with its coreceptors was also analyzed. The *TaBRI1* gene was overexpressed in Arabidopsis which led to faster germination, early flowering, and higher seed yield of the transgenic plants [58]. In barley a series of alleles of the homologous gene, *HvBRI1*, has been identified with the application of ‘forward-’ and ‘reverse-genetics’ approaches [2,12,59]. The mutations are localized in various fragments of this intronless gene and cause substitutions of amino acid residues located in various functional domains of the encoded BR receptor [2]. One of the alleles, *uzu1.a*, is a well-known source of semi-dwarfism in Northeast Asian short-culm cultivars and landraces [12]. Interestingly, it was reported that the semi-dwarf phenotype of the *uzu1.a* mutant is sensitive to elevated temperature (26 °C). At this temperature the *uzu1.a* mutant showed extreme dwarfing, in contrast with other semi-dwarf mutants of the *HvBRI1* gene. So, this effect is specific for the *uzu1.a* allele. Thus, it was concluded that the other alleles of the *HvBRI1* gene (without the temperature-sensitive phenotype) may be regarded as more reliable alternatives and sources of semi-dwarfism in the future barley breeding programs [2]. Some of the semi-dwarf mutants of the *HvBRI1* gene (including the *uzu1.a* mutant) were exposed to drought stress. Interestingly, under the control conditions the analyzed mutants showed significantly lower concentrations of the gibberellin GA_7_ and jasmonic acid (JA) when compared with the wild-type (reference) cultivar. This indicated that under the control conditions homeostasis of these phytohormones is dependent on the proper progress of the BR signaling. Importantly, the semi-dwarf BR mutants exhibited delayed wilting in reaction to the drought stress when compared with the wild-type cultivar [19]. The semi-dwarf mutants of the *HvBRI1* gene were analyzed to elucidate an impact of the disturbances in the BR signaling on accumulation of non-enzymatic antioxidants under the control and drought conditions. Interestingly, analysis of glutathione accumulation indicated that under the control conditions the BR-insensitive mutants contained significantly lower concentrations of this antioxidant when compared with the BR-biosynthesis mutants and the wild-type cultivar. Therefore, it was concluded that the BR sensitivity is required for normal accumulation of glutathione in barley [60] (Figure 1). Interestingly, it was reported that the *uzu1.a* mutant shows an enhanced resistance to a range of viral and fungal pathogens. It was postulated that the enhanced resistance results from a combination of native, constitutive and inducible defense responses [61]. 

The second component of the BR receptor complex in rice is encoded by the *OsBAK1* gene [41]. The rice genome contains four genes homologous to the *BAK1* gene in Arabidopsis, and *OsBAK1* is the closest homolog of the Arabidopsis counterpart [24,41]. Binding of the BR molecule to the OsBRI1 receptor kinase promotes its association with the OsBAK1 kinase and formation of the functional receptor complex (Figure 1). It is known that the activated receptor complex inactivates the OsGSK1 kinase (homolog of Arabidopsis BIN2), but the exact mechanism of the inactivation is yet unknown [24]. Loss-of-function mutation of the *OsBAK1* gene results in erect leaves and the BR insensitivity [47]. An antisense suppression of the *OsBAK1* gene resulted in the erect leaf phenotype of a rice line, but without any significant effect on plant height, reproduction and grain yield. Thus, the rice line may be considered as a potential candidate for breeding cultivars with the improved grain yield at high planting density [41]. Recently, a novel hypomorphic, semi-dwarf mutant of the *OsBAK1* gene with erect leaves, small grains, and a top-bending-panicle phenotype has been identified. The mutant phenotype results from an amino-acid substitution which leads to reduced stability of the OsBRI1-OsBAK1 heterodimer, and consequently, entails changes in plant height and panicle architecture [62]. A functional analysis of wheat homologs of the SERK family proteins (to which the BAK1 kinases belong) has been recently performed. Interestingly, wheat *SERK* transcripts show differences in their hormonal responsiveness—two of them are auxin-responsive, whereas accumulation of other three of the transcripts is regulated in a BR-specific manner. Overexpression of the *TaSERK* genes in Arabidopsis led to increased height and seed yield of transgenic plants. The results indicated that the *TaSERK* genes play redundant roles in maintaining plant development [63]. 

In rice, the BR signaling may be initiated in a way which is alternative to the one described above. The alternative pathway is mediated by the heterotrimeric G proteins (Figure 1). The G proteins are highly conserved transducers of various signals, including BR, GA, abscisic acid (ABA), and D-glucose, and are composed of three subunits: Gα, Gβ and Gγ [64]. Rice genome contains only one copy of each gene encoding the Gα and Gβ proteins and two genes encoding the Gγ protein [47]. The rice *D1* gene encodes the heterotrimeric G-protein alpha subunit (RGA1) which functions in several signaling pathways [53]. It was suggested that the Gα subunit functions separately from the canonical BR signaling pathway and promotes some of the BR responses through yet not fully identified downstream components. The canonical and the Gα-mediated signaling pathways act coordinately in some processes and independently in others [65]. Mutations of the *D1* gene lead to the characteristic BR-specific phenotype, including reduced height and erect leaves, which is caused by perturbation in the BR sensitivity, however, severity of the phenotype depends on a type of mutation. Interestingly, a feedback suppression of the BR-biosynthesis gene expression in response to exogenous BR application functions normally in the *d1* mutant (in contrast to the *d61* mutants of the *OsBRI1* gene in which the feedback mechanism is disturbed). This indicates that the OsD1/RGA1 protein in rice is involved in the distinct BR signaling relay, independent of the OsBRI1 receptor kinase [47,66,67,68]. It is known that mutation of the *OsD1/RGA1* gene affects gibberellin signal transduction and leads to hypersensitive response to a fungal infection [69,70]. This indicated that OsD1/RGA1 is involved in the GA signaling and disease resistance [50]. It was suggested that the OsD1/RGA1 protein may be also involved in perception of auxins and other signals. OsD1/RGA1 and OsBRI1 promote organ elongation by stimulation of cell divisions in an additive manner (parallel pathways). Moreover, OsD1/RGA1 may be involved in crosstalk between the BR and GA signaling pathways [47]. Interestingly, recent reports described mutants defective in two barley genes encoding the α-subunit and γ-subunit of the heterotrimeric G protein, *HvD1* and *HvDEP1*, respectively [65,71,72]. In a root growth-inhibition assay it was reported that the *brh1* mutant shows reduced sensitivity to BR [65]. Interestingly, it was reported that mutant alleles of the *Brh1* (*HvD1*) gene do not have any major negative impact on malting quality—a very important trait in barley breeding. Thus, it was concluded that the *brh1* alleles may be used as a source of semi-dwarfism for breeding of malting barley cultivars [72].

The signaling relay initiated by the OsD1/RGA1 protein is mediated in rice by the OsTUD1 (Taihu Dwarf1) U-box E3 ubiquitin ligase (also known as ELF1) which is localized to the plasma membrane (Figure 1). However, the OsTUD1/ELF1 protein does not appear to be ubiquitinated. Rice mutants of the *OsTUD1* gene show reduced BR sensitivity, which indicates that OsTUD1 is the BR signaling activator. Importantly, OsTUD1 is involved in the BR signaling but not in the gibberellin or cytokinin signaling. It was reported that the OsD1/RGA1 and OsTUD1 proteins directly interact to relay the BR signal which may be parallel or partly overlapping with the main OsBRI1-mediated pathway [50,73] (Figure 1). Recently, seven semi-dwarf mutants of the barley homolog of the *OsTUD1* gene, *Brh2*, which encodes the U-box E3 ubiquitin ligase have been identified. Interestingly, the barley mutants retained sensitivity to exogenous BR. The mutants exhibited erect phenotype, but grain yield of the mutants was reduced, particularly the 1000-grain weight was lowered [74].

In rice the Ca^2+^-dependent protein kinase (CDPK) and the Ca^2+^-dependent protein phosphorylation are involved in the BR-induced rice lamina inclination [75]. Perception of BR was shown to activate the membrane-bound mitogen-activated protein kinase (MAPK) and cytosolic CDPK protein, but this effect was reversed in the mutant plants defective in the OsBRI1 function. It indicated that the signaling pathways downstream of the BR-mediated activation of MAPK and CDPK are parallel, and may involve other components in addition to OsBRI1 to mediate the BR response [76] (Figure 1).

It was recently reported that the early stages of the BR signaling in rice are modulated by the small GTPase, OsPRA2, which binds the OsBRI1 receptor kinase at the plasma membrane. The OsPRA2 protein is a negative regulator of the BR signaling initiation as it represses the OsBRI1 kinase activity and its interaction with the OsBAK1 kinase (Figure 1). The OsPRA2 activity leads to reduced sensitivity to the BR treatment [51]. The OsPRA2 protein interacts with another novel component regulating the BR signaling initiation in rice—the OsGAP1 protein (GTPase Activating Protein1). Overexpression of the *OsGAP1* gene leads to the BR-deficiency phenotype. It indicates that OsGAP1 stimulates the GTPase activity of OsPRA2 which is important for the repression of the BR signaling initiation [25,77] (Figure 1). 

Function of the OsBRI1 receptor in rice is also regulated by the OsELT1 (Enhanced Leaf inclination and Tiller number1) receptor-like protein. A knock-out mutation or the RNAi-mediated silencing of the *OsELT1* gene resulted in BR insensitivity, and consequently, in reduction of plant height, leaf inclination and tiller number. The *OsELT1* gene encodes a transmembrane protein which stimulates the BR signaling by inhibiting ubiquitination and subsequent endocytosis-mediated degradation of the OsBRI1 receptor (Figure 1). The intracellular domain of the OsELT1 protein interacts specifically with the intracellular domain of the OsBRI1 receptor kinase, but does not exhibit kinase activity. Importantly, the OsELT-OsBRI1 interaction does not influence the interaction between the OsBRI1 and OsBAK1 kinases. The ELT1 protein and its positive effect on the BR signaling is probably specific to monocots [36].

## 3. Regulation of the BR Signaling Relay in Monocots

In rice, the BR perception by the OsBRI1-OsBAK1 receptor complex initiates a signaling cascade which is conveyed to the OsBSK3 kinase (BR Signaling Kinase3) (Figure 1). OsBSK3 belongs to the RLCK (Receptor-Like Cytoplasmic Kinases) family of proteins, and apart from the kinase domain, contains a C-terminal TPR (Tetratricopeptide Repeat) domain. The TPR domain of the OsBSK proteins functions as a phosphorylation-dependent autoinhibitory domain to control the BSK protein activity. It was reported that OsBSK3 is a positive regulator of the BR signaling in rice and is directly bound and phosphorylated by the OsBRI1 receptor kinase. The TPR domain of OsBSK3 interacts with its kinase domain to prevent interaction of OsBSK3 with downstream components of the BR signaling in the absence of BR perception. The OsBRI1-mediated phosphorylation of OsBSK3 disrupts the interaction between the TPR and kinase domains of this protein and enables the interaction of OsBSK3 with downstream components of the BR signaling [28] (Figure 1). 

Upon the BR signaling initiation, the OsBSK3 protein phosphorylates/activates an unidentified phosphatase and consequently represses the cytoplasmic OsGSK (Glycogen Synthase Kinase) proteins, mainly the OsGSK2 kinase [28,48,78]. The OsGSK1 and OsGSK2 kinases are negative regulators of the BR signaling relay in rice [42,47,48] (Figure 1). Loss-of-function mutation of the *OsGSK1* gene results in enhanced BR response [42,47], however, OsGSK2 plays a major role in regulating the BR response through phosphorylation-mediated suppression of a group of proteins, including the key transcription factors OsBZR1, OsLIC and OsDLT [48] (Figure 1), which will be described below. Rice genome contains nine genes encoding the OsGSK proteins, and four of them show a high sequences similarity with the BIN2 kinase of Arabidopsis [79,80]. It is suggested that the rice GSK proteins may play redundant roles in the BR responses [47,48]. Interestingly, one of the rice QTLs (Quantitative Trait Loci) named Thousand Grain Weight3 controls rice grain size and encodes the OsGSK5 kinase. However, OsGSK2 and OsGSK5 play different roles in the BR signaling [81].

Apart from the involvement in the BR signaling, OsGSK1 may also participate in stress responses. A knockout mutant of the *OsGSK1* gene obtained through T-DNA insertion showed enhanced tolerance to abiotic stresses, such as cold and heat stress, salinity, and drought. It indicated that OsGSK1 kinase may play a major role in regulation of reaction of rice plants to the abiotic stresses [24,42]. The OsGSK1 protein may control the abiotic stress tolerance through suppression of stress responsive genes [42]. However, in the BR signaling pathway the major role of the OsGSK2 kinase is phosphorylation and inhibition of activity of the following transcription factors (described below): OsBZR1, OsLIC, OsDLT, OsRLA1/SMOS1, OsOFP1, and OsOFP8 (Figure 1) which are crucial for the regulation of the BR-dependent gene expression [24,37,38,48,49,82,83]. In rice (and Arabidopsis) the GSK proteins regulate in the phosphorylation-dependent manner various substrate proteins which represent different signaling and developmental processes. This allows the GSK proteins to perform the role of master regulators influencing downstream effects of several signaling pathways, apart from being crucial modulators of the BR response [48]. Recently, a bioinformatics analysis indicated that the barley genome contains seven transcriptionally active *GSK* genes which are expressed ubiquitously, at different developmental stages, and encode highly conserved proteins [84]. 

It was recently reported that natural variations of OsGSK2 which alter its kinase activity determine rice mesocotyl elongation. BRs stimulate the mesocotyl elongation by repressing the OsGSK2-mediated phosphorylation of the U-type cyclin CYC U2 which controls cell division. The BR signaling promotes mesocotyl elongation in rice primarily by stimulating the cell division. The OsGSK2-mediated phosphorylation reduces the CYC U2 protein stability and consequently represses cell division in the mesocotyl. On the other hand, the mesocotyl elongation in rice is negatively regulated by another phytohormone, strigolactone (SL), as the F-box D3 (Dwarf3) protein (which is the major positive regulator of the SL signaling) interacts with the phosphorylated CYC U2 cyclin to initiate its ubiquitination-mediated degradation (Figure 1). This result shed some light on the rice domestication process—it was suggested that natural alleles of the *OsGSK2* gene were selected to regulate the rice mesocotyl length by coordinating the BR and SL signaling pathways [85]. The OsGSK2 kinase may also constitute a point of interactions between the BR and auxin signaling pathways, as it was reported that it interacts with the OsARF4 (Auxin Response Factor4) and AP2 (Apetala2) transcription factors [37,81]. It was recently hypothesized that these transcription factors are negatively regulated by OsGSK2 (Figure 1) and play parallel role with OsBZR1 [78].

Another regulative component of the BR signaling in rice has been recently identified. A quantitative trait locus, *qGL3* (Grain Length3), encodes a protein phosphatase containing the Kelch-like repeat domains—OsPPKL1 (Protein Phosphatase Kelch-Like1). Overexpression of the *qGL3* gene resulted in the BR-deficiency phenotypes. OsPPKL1 interacts directly with the rice OsGSK3 kinase and dephosphorylates it. Interestingly, in contrast to dephosphorylation of Arabidopsis BIN2 kinase which results in the BIN2 degradation, in rice OsPPKL1 dephosphorylates and stabilizes the OsGSK3 protein. Therefore, OsPPKL1 is a negative regulator of the BR signaling in rice, as it enhances stability of the OsGSK3 which in turn regulates phosphorylation status and subcellular distribution of the OsBZR1 transcription factor (Figure 1). Apart from role of the OsPPKL1 phosphatase in regulation of grain length in rice its function in the OsGSK3 stabilization constitutes a significant difference in the BR signaling model between rice and Arabidopsis. It is hypothesized that different phosphorylation sites in Arabidopsis BIN2 and rice OsGSK3 may be responsible for the opposite degradation patterns. In rice there are three homologs of the OsPPKL proteins [78]. Interestingly, OsPPKL1 and OsPPKL3 play a negative role in the regulation of grain length, whereas OsPPKL2 plays a positive role in this process [86].

The OsGSK2 protein plays also an important role in regulation of grain size and yield in rice. It was reported that the rice GL2/OsGRF4 (Grain Length2/Growth Regulation Factor4) protein, apart from being involved in the BR response, affects grain size. OsGSK2 interacts with the GL2/OsGRF4 protein to repress its activity as a transcription factor enhancing gene expression to regulate grain length [87]. On the other hand, the OsGSK2 kinase activity may be suppressed by another factor - GW5 (Grain Width5). GW5 is a calmodulin-binding protein which is expressed predominantly in panicles and strongly affects grain width, weight, and lamina bending in rice. The GW5 protein is localized to the plasma membrane and directly interacts with the OsGSK2 kinase to repress its activity (Figure 1), which results in enhanced accumulation of dephosphorylated transcription factors OsBZR1 and OsDLT (described below) in the nucleus to regulate the BR-dependent gene expression. The GW5 protein represses both auto- and transphosphorylation activity of the OsGSK kinase. Thus, GW5 is a novel positive regulator of the BR signaling pathway in rice [44]. Recently, a novel homolog of the *GW5* gene in rice, *GW5L* (GW5-Like), has been identified. Subcellular localization, biochemical function and positive role of the GW5L protein in the BR signaling regulation are similar to those of the domestication-related GW5 homolog [88]. Moreover, the GW5 and GW5L proteins may also be involved in the salt-stress reaction through interaction with the calmodulin protein OsCaM1-1 which is a positive regulator of the salt stress tolerance [88,89]. It was suggested that GW5 and GW5L may directly or indirectly repress transcription of the *OsCaM1-1* gene. Therefore, both GW5 and GW5L may act as regulators of grain size and the salt-stress tolerance in rice (Figure 1). It was suggested that the GW5 and GW5L homologs may serve as candidates for genetic manipulation through targeted genome editing to improve grain yield and salt-stress tolerance in rice and perhaps in other cereals [44,88].

Recently, a novel component of the BR signaling which acts as a regulator of the BIN2 kinase homolog in sorghum (*Sorghum bicolor*) has been identified. Mutation of the sorghum *DW1* (Dwarfing1) gene led to the BR deficiency-specific phenotype, including erect growth habit, defect in skotomorphogenesis, and the BR insensitivity. The DW1 protein interacts with the sorghum homolog of the BIN2 kinase and inhibits its nuclear localization. Thus, it was concluded that DW1 protein positively regulates the BR signaling by repressing function of the BIN2 kinase. The activity of the DW1 protein allows for a fine-tuning of the BR signaling through the inactivation of the BIN2 homolog [90] (Figure 1). 

## 4. Regulation of the BR-Dependent Gene Expression in Monocots

**Figure 2:**
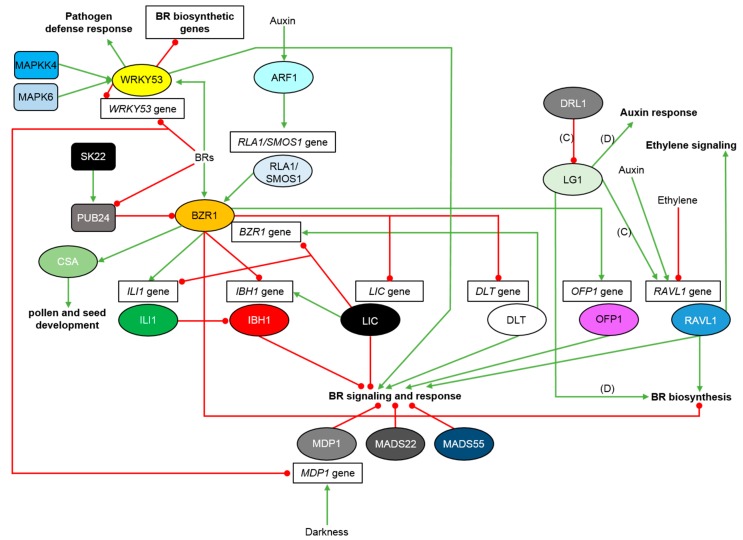
Molecular mechanisms of regulation of the BR-dependent gene expression in rice and other monocots. Transcription factors are shown as ovals, other proteins are depicted as rectangles. Green arrows indicate stimulation, whereas red lines with the bullet points represent suppression. Detailed description is given in the text. Some of the interactions and responses were described in maize (**C**) and wheat (**D**).

The OsBZR1 transcription factor in rice is the closest ortholog of the BZR1 and BES1 transcription factors from Arabidopsis and functions as a positive regulator of the BR signaling (Figure 1). The rice genome contains four genes encoding homologous OsBZR1 proteins. The RNAi-mediated silencing of the *OsBZR1* gene expression results in the BR insensitivity, semi-dwarfism and erect phenotype [24,47]. It was also reported that in rice the OsBZR1 transcription factor directly promotes expression of the *CSA* (Carbon Starved Anther) gene which encodes a MYB-domain transcription factor. The CSA protein is involved in a direct activation of expression of genes regulating sugar partitioning, starch synthesis and metabolism during pollen and seed development. Thus, it was suggested that BRs promote rice pollen grain formation and seed development in the OsBZR1-dependent manner [91] (Figure 2). As mentioned above, OsBZR1 is phosphorylated by the OsGSK kinases, and similarly to Arabidopsis, the phosphorylated form of the OsBZR1 transcription factor is bound by the 14-3-3 proteins and retained in the cytoplasm [43]. Interestingly, accumulation of variants of the OsBZR1 protein is developmentally regulated—the phosphorylated form is not present in young leaves, whereas it becomes accumulated in mature leaves [48]. Despite its functional importance, the mechanism controlling the OsBZR1 stability has not been fully understood. However, it was recently reported in rice that the U-box E3 ubiquitin ligase OsPUB24 acts as a negative regulator of the BR signaling through ubiquitination and subsequent proteasome-mediated degradation of OsBZR1. Stability of the OsPUB24 protein is repressed by BRs. Interestingly, a rice homolog of the Arabidopsis BIN2 kinase, OsSK22, was reported to phosphorylate the OsPUB24 protein to enhance its stability (Figure 2). Therefore, it was concluded that the OsPUB24 ubiquitin ligase participates in OsBZR1 turnover, and that the regulatory mechanism mediated by the OsPUB24 and OsSK22 proteins is pivotal for modulating the BR signaling in rice [92]. Recently, a bioinformatics and functional analysis of putative *BZR1* homologs in the maize (*Zea mays*) genome has led to identification of eleven *ZmBZR1* genes. It was suggested that expansion of the *BZR1* genes in the maize genome occurred after divergence of monocots and dicots. It was reported that promoter regions of the *ZmBZR1* genes contain numerous light-responsive and ABA-responsive elements. Five of the genes did not show transcript accumulation which indicated that expression of the genes may be developmentally regulated. Expression of the other six genes was ubiquitous. Moreover, the *ZmBZR1* genes showed differential expression pattern in response to abiotic stresses and light signal [93,94].

Activity of OsBZR1 is also modulated by the RLA1/SMOS1 (Reduced Leaf Angle1/Small Organ Size1) transcription factor which contains the APETALA2 DNA-binding domain. RLA1/SMOS1 functions as a positive regulator in the BR signaling (Figure 1) and is required for the OsBZR1 function. The RLA1/SMOS1 protein interacts with OsBZR1 to promote its transcriptional activity to cooperatively regulate downstream genes (Figure 2). 

As mentioned above, function of RLA1/SMOS1 is regulated by the OsGSK2 kinase which interacts with and phosphorylates RLA1/SMOS1 to reduce its stability [37]. Moreover, RLA1/SMOS1 may constitute a novel integration node in the crosstalk between the BR and auxin signaling pathways, as it is known that auxin may induce the *RLA1/SMOS1* gene transcription through OsARF1-mediated direct binding to its promoter [37,95] (Figure 2).

In rice OsBZR1 directly regulates yield-determining genes which influence various physiological processes [38]. It was reported that OsBZR1 directly regulates expression of two genes encoding other transcription factors—OsILI1 (Increased Leaf Inclination1) and OsIBH1 (ILI1-binding bHLH protein1). However, OsBZR1 influences expression of the *OsILI1* and *OsIBH1* genes in an opposite manner: OsBZR1 stimulates expression of the *OsILI1* gene, but suppresses expression of the *OsIBH1* gene (Figure 2). OsILI1 and OsIBH1 interact with each other to antagonistically regulate the BR signaling and the leaf inclination in rice [96,97]. OsILI1 belongs to the HLH (helix-loop-helix) subfamily of transcription factors which lack the basic domain required for DNA binding. However, the HLH transcription factors may dimerize with the DNA-binding basic HLH (bHLH) transcription factors to regulate expression of target genes. It is known that OsILI1 directly interacts with the OsIBH1 transcription factor and suppresses its activity to regulate cell elongation. The OsIBH1 transcription factor belongs to the bHLH group and acts as a negative regulator of the BR response. In this model of signaling, which is conserved in rice and Arabidopsis, the BR-upregulated HLH protein OsILI1 inactivates through heterodimerization the bHLH negative regulator OsIBH1. Thus, BRs suppress the OsIBH1 function at both transcriptional and posttranslational level [47] (Figure 2). This antagonistic activity of transcription factors makes them a potential candidates for modification to design optimal architecture of rice plants [24,49,96]. 

Another regulator of the BR signaling in rice is a CCCH-type transcription factor OsLIC. Antisense suppression of the *OsLIC* gene resulted in enlarged leaf and tiller angles and decreased number of grains per panicle. On the other hand, gain-of-function mutation of the *OsLIC* gene led to erect phenotype and decreased BR sensitivity. Activity of the OsLIC transcription factor is regulated in a similar manner as of OsBZR1: OsLIC interacts with OsGSKs and is phosphorylated by these kinases, which results in retention of OsLIC in the cytoplasm (Figure 1). The BR perception inhibits the OsGSK activity what results in accumulation of unphosphorylated form of the OsLIC proteins and their transport to the nucleus. Then, OsLIC directly binds to promoter of the *OsBZR1* gene to suppress its expression. Apart from repressing the *OsBZR1* gene transcription, it was also reported that OsLIC has a negative effect on transcription of the *OsILI1* gene. Interestingly, OsLIC binds the *OsIBH1* promoter to enhance expression of the gene and balance the opposite effect mediated by OsBZR1. On the other hand, the *OsLIC* gene is a direct target of the OsBZR1 transcription factor which represses the *OsLIC* gene expression. Thus, the OsLIC and OsBZR1 transcription factors act antagonistically, mutually repressing their expressions at the transcriptional level (Figure 2). This antagonistic mechanism regulates cell elongation, and consequently, leaf inclination in rice. OsLIC is a significant negative regulator of the BR response functioning downstream of the OsGSK kinases [24,49] (Figure 1 and Figure 2). However, an explanation of the role of these transcription factors stems from their differential expression profile at various BR concentrations. OsBZR1 is active at low BR concentration to stimulate the BR signaling, whereas OsLIC is activated at high BR concentration to dampen the BR signaling [49]. Therefore, in rice (and potentially in other monocots) the BR-dependent gene expression is modulated based on equilibrium between the OsBZR1 (positive regulator) and OsLIC (negative regulator) activities which are regulated at the transcriptional and translational levels [24,49,98]. In rice OsLIC interacts with and antagonizes OsBZR1 to negatively regulate the BR response [49] (Figure 2). 

Another regulator of the BR-dependent gene expression is the OsDLT protein which belongs to the plant-specific GRAS family of transcription factors. Mutation of the *OsDLT* gene (T-DNA insertion) resulted in the BR insensitivity-related phenotype, including semi-dwarfism, erect leaves and reduced tiller number. It was reported that OsDLT positively regulates the BR response in rice, but it acts downstream of both OsBRI1 and OsGSK2 in the BR signaling pathway [45,48]. OsDLT is another direct substrate of the OsGSK2 kinase—OsGSK2 interacts with and phosphorylates OsDLT [48,99] (Figure 1). However, it was reported that OsGSK2 phosphorylates OsBZR1 more preferably than OsDLT. The BR perception promotes accumulation of dephosphorylated form of OsDLT via inhibition of the OsGSK2 kinase activity. Similarly to OsBZR1, accumulation of the OsDLT protein is also developmentally regulated—phosphorylated form of OsDLT accumulates preferably in mature organs. In rice seedlings most of the OsBZR1 and OsDLT proteins are in the active (dephosphorylated) forms which function to promote plant growth [48]. However, expression of the *OsDLT* gene is repressed by BRs at the transcriptional level in the OsBZR1-mediated manner, on the basis of feedback mechanism [45,48]. Interestingly, OsBZR1 and OsDLT regulate each other in an opposite way: OsBZR1 represses the *OsDLT* gene expression, whereas OsDLT promotes expression of the *OsBZR1* gene (Figure 2). Thus, it was concluded that modulation of the BR responses requires a complex regulatory network [99]. Moreover, OsDLT is involved (in concert with OsBZR1) in the feedback inhibition of the BR biosynthetic genes [45]. It is interesting that BRs have a dual and opposite effect on OsDLT: BRs repress transcription of the *OsDLT* gene, but promote the OsDLT protein activity by enhancing its dephosphorylation. In general, OsBZR1 and OsDLT positively regulate the BR signaling and response in rice, whereas OsLIC is a negative regulator of these processes [24,45,48] (Figure 1 and Figure 2). It was postulated that BR, in the OsBZR1-dependent manner, regulates rice tillering through the control of the *OsDLT* gene transcription [24]. Since OsDLT and OsBZR1 cannot directly interact with each other [48], it was recently suggested than an adaptor may be involved to mediate their interaction. The aforementioned OsRLA1/SMOS1 transcription factor may play a role of the adaptor to mediate the formation of the OsBZR1-RLA1-DLT complex, which can be phosphorylated by the OsGSK2 kinase [37,100].

Recently, the OsOFPs (Ovate Family Proteins) transcription factors which interact with OsDLT have been identified in rice [82,83]. It should be noted that the rice genome contains 31 *OsOFP* genes and their functions in plant development are not fully understood [82]. The *OsOFP1* gene is ubiquitously expressed and transcription of the gene is greatly promoted by BR in the OsBZR1-dependent manner (Figure 2). It was reported that the OsOFP1 protein directly interacts with the OsGSK2 kinase which represses the OsOFP1 protein accumulation and activity (Figure 1). The inhibitory effect of the OsGSK2 kinase is attenuated by BRs which enhance the OsOFP1 protein stability. Interestingly, overexpression of the *OsOFP1* gene resulted in a decreased transcription of the GA biosynthetic genes, whereas expression of the GA inactivation gene was increased. It indicates that OsOFP1 transcription factor is involved in the BR-induced repression of the GA accumulation (Figure 1), however, this process is dependent on the BR concentration, and tissue-specific. The OsOFP1 protein plays a positive role in regulating the BR signaling (Figure 1 and Figure 2) to influence plant architecture and grain morphology in the OsDLT-dependent manner [83]. A homolog of the OsOFP1 transcription factor, OsOFP8, also plays a positive role in regulating the BR signaling. It was reported that BRs induce expression of the *OsOFP8* gene and stimulate accumulation of the encoded protein. Interestingly, the BR concentration (1 µM) which enhanced the *OsOFP8* transcript accumulation caused a decrease in accumulation of the *OsBZR1* and *OsDLT* transcripts, which indicates a differential BR-dependent regulation of expression of these genes. Overexpression of the *OsOFP8* gene repressed expression of the BR biosynthetic gene *OsD2*, but increased expression of the *OsBZR1* gene. Similarly to OsOFP1, the OsOFP8 protein interacts with and is phosphorylated by the OsGSK2 kinase. It was reported that the OsOFP8 protein interacts specifically with the OsGSK2 kinase, but not with the OsBZR1 or OsDLT transcription factors. The phosphorylated form of OsOFP8 is exported from the nucleus to the cytoplasm and degraded [82] (Figure 1).

Another rice gene, *OsBU1* (BR Upregulated1), encodes a HLH transcription factor which is a positive regulator of the BR signaling. Interestingly, OsBU1 constitutes a point of convergence of the signaling pathways initiated by OsBRI1 and OsD1/RGA1 [67,68,101] (Figure 1). Loss-of-function mutation of the *OsBU1* gene results in erect leaves. Therefore, it was suggested that OsBU1 controls leaf inclination downstream of both OsBRI1 and OsD1/RGA1 pathways. The *OsBU1* gene is highly expressed in the lamina joint and in panicle, particularly at the heading stage. Transcription of the *OsBU1* gene is not influenced by auxins or gibberellin, however, it is suppressed by ABA and greatly stimulated by exogenous BR as a primary BR-signaling response gene [101] (Figure 1). The BR-dependent stimulation of *OsBU1* expression is mediated by both OsBRI1 and OsD1/RGA1 proteins. As the OsBU1 transcription factor lacks the basic domain required for DNA binding and belongs to the HLH family [102], it functions through interactions with other transcription factors [101]. The positive role of OsBU1 in the BR signaling is probably mediated through inhibition of a transcription factor (maybe a bHLH protein) which functions as a negative regulator of the process [47]. Regulating the expression of the *OsBU1* gene is considered as a method to increase grain yield in rice [101]. 

Recently, a novel homolog of the *OsBU1* gene, *OsBUL1* (Brassinosteroid Upregulated Like1), has been identified as a positive regulator of lamina inclination and grain size in rice [103]. Moreover, based on an analysis of the cDNA microarray data another novel component of the pathway, OsBDG1 (BUL1 Downstream Gene1) has been identified as acting downstream of OsBUL1. The *OsBDG1* gene encodes a small protein which belongs to the LRR (Leucine-Rich Repeats) family. Overexpression of the *OsBDG1* gene resulted in increased leaf angle and grain size, whereas the RNAi-mediated silencing of the gene led to erect plant phenotype. Transcription of the *OsBDG1* gene is enhanced in response to BR. Interestingly, the OsBDG1 protein upregulates the *OsAP2* and *OsWRKY24* genes, which encode transcription factors, in lamina joints. These transcription factors are positive regulators of leaf inclination and grain size in rice [104].

Another component of the BR signaling in rice, OsRAVL1 (Related to ABI3/VP1 RAV-Like1), is a B3-domain transcription factor. Mutants of the *OsRAVL1* gene exhibited reduced BR sensitivity and the semi-dwarf phenotype. OsRAVL1 positively regulates expression of the BR receptor gene *OsBRI1* and the BR biosynthetic genes *OsD2*, *OsD11* and *OsBRD1* through direct binding to promoters of these genes. It was shown that expression of the *OsBRD1* gene is inhibited by the OsBZR1 transcription factor (mediating the negative BR feedback response) and that OsBZR1 dominates over OsRAVL1 during controlling the *OsBRD1* gene transcription. In rice, BR biosynthesis is controlled by positive and negative factors. Thus, OsRAVL1 is a key regulator of the BR homeostasis in rice which ensures a basal activity of both BR signaling and biosynthesis pathways. Interestingly, expression of the *OsRAVL1* gene is not controlled by exogenous BR [46]. However, it was reported that transcription of the *OsRAVL1* gene is induced by the 2,4-D (synthetic auxin) treatment, but it is repressed by the ACC (1-Aminocyclopropane-1-carboxylic acid which is the ethylene precursor) application. Apart from the positive effect on the BR signaling, RAVL1 was also reported to activate the ethylene signaling in rice (Figure 2). Thus, suppression of the *OsRAVL1* expression by the ACC application might be a part of negative feedback mechanism. This indicates that OsRAVL1 may constitute a point of crosstalk between these phytohormones [105]. However, it should be kept in mind that ACC may also act independently on ethylene. As it is currently unknown whether the *OsRAVL1* gene is controlled by ethylene and/or ACC, the possible ethylene-independent action should not be excluded and a further research is needed to fully clarify the issue. Recently, a QTL, *UPA2* (Upright Plant Architecture2), which causes reduced leaf angle, and consequently more upright plant stature has been identified in maize. The QTL harbors a 2-bp deletion upstream of the maize *RAVL1* homolog—*ZmRAVL1*. Interestingly, the 2-bp deletion differentiates the modern maize cultivars from the wild relative—teosinte. The B3-domain transcription factor encoded by the *ZmRAVL1* gene is a positive regulator of leaf inclination in maize, as it directly activates the BR biosynthetic gene *ZmBRD1*. Moreover, a mechanism of the *ZmRAVL1* gene expression regulation has been described. Transcription of the *ZmRAVL1* gene is activated by the LG1 (LIGULELESS1) transcription factor which binds directly to the *ZmRAVL1* promoter. The LG1 protein is bound by another transcription factor, DRL1 (Drooping Leaf1) [5,6]. It was reported that DRL1 has a zinc-finger DNA binding domain, and its loss-of-function mutation leads to increased leaf angles [106]. The DRL1 protein interacts directly with the LG1 transcription factor to repress its transcriptional activation of the *ZmRAVL1* gene (Figure 2). The above-described 2-bp deletion present in the upstream region of the *ZmRAVL1* gene in the modern maize cultivars reduces the interaction and inhibitory effect of the DRL1 transcription factor on the LG1 protein, and consequently leads to increased expression of the *ZmRAVL1* gene and the BR biosynthesis. It was suggested that the *ZmRAVL1* gene may serve as a target for genetic manipulation of the leaf angle and the erect maize plant architecture in the future breeding programs [5,6]. Recently, a wheat homolog of the *LG1* gene, *TaSPL8* (Squamosa Promoter Binding-Like8) has been identified. A knock-out mutation of the *TaSPL8* gene resulted in erect leaves, a compact architecture, and increased spike number especially under the high-density planting conditions. The encoded TaSPL8 protein activates genes related to the auxin response (*TaARF6*), the BR biosynthesis (*TaCYP90D2*), and cell elongation. Consequently, it regulates leaf inclination in wheat (Figure 2). Therefore, it was concluded that the *TaSPL8* gene may be regarded as a potential target for modulation of the plant architecture in cereals [8].

Recently, another transcription factor, OsWRKY53, which plays a role of positive regulator of the BR signaling in rice has been identified [107]. The WRKY proteins play various roles in plant reactions to biotic and abiotic stresses, as well as regulate diverse physiological processes through modulations of target gene expression [108]. A mutant of the *OsWRKY53* gene exhibited erect leaves and smaller grains, whereas overexpression of the gene resulted in enlarged leaf angles and grain size [107]. Interestingly, apart from regulating plant architecture, the OsWRKY53 transcription factor positively regulates pathogen defense response [109]. The OsWRKY53 transcription factor interacts with and is phosphorylated by the OsMAPKK4-OsMAPK6 proteins, and the phosphorylation is crucial for the OsWRKY53 function in the regulation of BR response [107]. Mutation of either the *OsMAPKK4* or *OsMAPK6* gene results in the BR-deficient phenotypes (dwarfism, erect leaves and small size of grains) and impaired BR signaling [110,111]. The OsWRKY53 transcription factor acts downstream of the main, OsBRI1-mediated BR signaling pathway. It was reported that BRs have a repressive effect on the *OsWRKY53* transcription, but stimulatory impact of the OsWRKY53 protein accumulation. Moreover, OsWRKY53 participates in the feedback inhibition of the BR biosynthetic genes, and may suppress its own expression [107] (Figure 2). 

In rice, the BR signaling and leaf inclination are regulated by three genes encoding the SVP (Short Vegetative Phase)-group MADS-box transcription factors OsMADS47/OsMDP1 (MADS-domain-containing protein1), OsMADS22 and OsMADS55. These proteins are negative regulators of the BR signaling [112,113]. It was reported that transcription of the *OsMDP1* gene is stimulated by darkness but suppressed by the BR treatment (Figure 2). Deficiency of the *OsMDP1* gene resulted in altered expression of numerous genes which encode proteins involved in various processes, such as cyclins, proteins involved in transcription and signaling, and hormone (mostly auxin) function. Therefore, it was concluded that OsMDP1 plays versatile roles in plant development and responses to light and BRs [112]. It was reported that OsMADS55 works as a major negative regulator of the BR responses, whereas OsMADS22 plays a supportive role. All the above MADS proteins act as negative regulators of the BR responses, but their spatial and temporal roles are diversified [113].

## 5. Hormonal Signaling Crosstalk and Its Role in Regulation of Physiological Processes in Monocots

**Figure 3:**
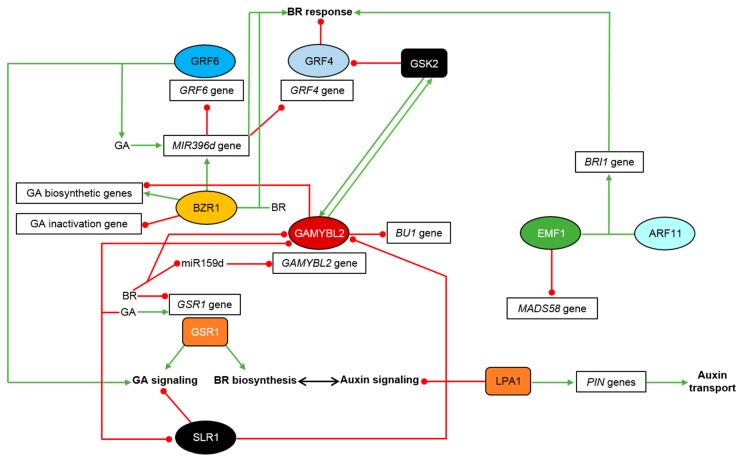
Molecular mechanism of crosstalk between the BR, auxin and GA signaling pathways which enables regulation of various physiological and developmental processes in rice and other monocots. Transcription factors are shown as ovals, other proteins are depicted as rectangles. Green arrows indicate stimulation, whereas red lines with the bullet points represent suppression. Detailed description is given in the text.

Interactions of the BR signaling with signaling pathways of other phytohormones regulate plant yield and stress responses [114]. Moreover, the signaling pathways of BR, GA and auxin, and their crosstalk, play an important role in regulation of leaf inclination in rice [115]. Biosynthesis and signaling processes of BR and GA interact to form a complex network [49,116]. However, in monocots molecular mechanisms of the interplay between the GA and BR signaling pathways remain largely unknown [117], although the crosstalk between these two phytohormones in regulation of various physiological processes was reported [116,118]. In rice the OsGSR1 protein constitutes one of the points of interaction between the GA and BR response pathways. The OsGSR1 protein belongs to the GAST (GA-stimulated transcript) family. It was reported that expression of the *OsGSR1* gene is induced by GA, but repressed by BR (Figure 3). Interestingly, a transgenic rice line in which expression of the *OsGSR1* gene was reduced by RNAi showed phenotypes similar to the BR-defective mutants (including the dwarfism and erect leaves). Moreover, the RNAi-mediated silencing of the *OsGSR1* gene led to higher accumulation of the endogenous GA, and reduced sensitivity to GA. The OsGSR1 protein stimulates the BR biosynthesis through a direct regulation of the BR biosynthetic DIM/DWF1 enzyme at the post-translational level. The OsGSR1 interacts with the BR biosynthetic enzyme and in this way regulates the BR biosynthesis in rice (Figure 3). The *OsGSR1* RNAi transgenic plants showed higher expression of the *OsBRI1* and *OsDWARF* genes, which is consistent with reduced BR signaling resulting from the decreased concentration of the endogenous BRs. OsGSR1 is a positive regulator of the GA signaling, and regulates the BR biosynthesis at the posttranslational level. Therefore, it constitutes a point of interaction between the GA and BR metabolic pathways [116] (Figure 3). The rice BR-GA hormonal crosstalk may also be illustrated by the fact that OsBZR1 directly binds to promoters of the *Ga20ox-2*, *GA3ox-2* (GA biosynthetic genes) greatly inducing their expression, and the *GA2ox-3* (GA inactivation gene) repressing its transcription (Figure 3). Generally, BR promotes GA biosynthesis and inhibits GA inactivation. BR signaling components are involved in both processes. As a feedback mechanism, GA extensively inhibits BR biosynthesis and the BR response in rice [10,118]. Interestingly, exogenous application of BR at high concentrations suppresses the GA biosynthesis. This may partially explain the surprising phenomenon that the high BR doses may inhibit plant growth [10,38].

The interplay between the BR and GA signaling pathways may also be mediated by microRNAs. MicroRNAs participate also in the hormone-dependent regulation of agricultural traits [119]. It was reported that miR396 represents one of the most conserved miRNA families in plants [120]. In rice OsmiR396 targets the *GRFs* (Growth Regulating Factors) genes to regulate pathways of several phytohormones, such as BR, GA, and auxin [87,121,122], and in this way influences several processes, including leaf development, plant height and architecture, flowering time and seed size [87,122,123,124,125,126]. It was predicted that there are 12 *OsGRF* target genes of OsmiR396d in the rice genome which regulate various developmental processes. In rice, OsmiR396d is involved in the BR-GA signaling crosstalk. Overexpression of the *OsMIR396d* gene results in the BR signaling enhancement, but in suppression of the GA biosynthesis and signaling processes. *OsMIR396d* is involved in the regulation of GA signaling by repression of the *OsGRF6* gene expression, and this activity is independent of the BR signaling. It was reported that *OsMIR396d* exerts a positive effect on several steps of the BR signaling pathway in rice. Transcription of the *OsMIR396d* gene is stimulated by BRs and is directly promoted by the OsBZR1 transcription factor (Figure 3). Interestingly, transcription of the *OsMIR396d* gene is also stimulated by high concentrations of GA which indicates that OsmiR396d may participate in a feedback regulation of the GA biosynthesis. OsBZR1 enhances expression of the *OsMIR396d* gene to suppress expression of the *OsGRF6* gene which is involved in positive regulation of the GA biosynthesis and signaling, and consequently plant height [127] (Figure 3). It was reported that OsmiR396d participates also in the regulation of rice leaf inclination by targeting and repressing expression of the *OsGRF4* gene which suppresses the BR responses in the lamina joint bending [87,126]. Interestingly, activity of the OsGRF4 transcription factor is inhibited by direct binding of the OsGSK2 kinase [87] (Figure 3). Thus, the *OsMIR396d* gene is regulated by BR and GA and influences, in an opposite manner, two key agricultural traits—plant height (negatively) and leaf inclination (positively) through different downstream targets [127]. It was also recently suggested that the enhanced BR signaling induces the GA inactivation in reproductive tissues to suppress plant height [38].

A recent report indicated that the BR-GA crosstalk is mediated by another highly conserved microRNA—OsmiR159d. It was shown that BR suppresses expression of OsmiR159d which represses the target *OsGAMYBL2* gene encoding a transcription factor. Thus, the BR application results in a rapid decrease in the OsmiR159d accumulation and increase in the *OsGAMYBL2* transcript accumulation. On the other hand, the BR application induced degradation of the OsGAMYBL2 protein (Figure 3). The OsmiR159d-mediated inhibition of the *OsGAMYBL2* function represents an early BR response and regulates expression of the aforementioned *OsBU1* gene (which encodes the component of the BR signaling), as well as two genes involved in the GA biosynthesis. The OsGAMYBL2 transcription factor directly represses transcription of the *OsBU1* gene. Interestingly, the OsGSK2 kinase interacts with and phosphorylates the OsGAMYBL2 protein to prevent its degradation upon BR application. Moreover, the interaction with OsGAMYBL2 stabilizes the OsGSK2 kinase upon the BR application (Figure 3), which results in suppression of the BR signaling. The OsGAMYBL2 transcription factor represses transcription of the GA biosynthetic genes. Interestingly, the GA application results in degradation of the OsGAMYBL2 protein [117]. It is also known that the GA application leads to a rapid degradation of the SLR1 (Slender Rice1) protein which belongs to the DELLA family of negative regulators of the GA signaling [128,129]. On the other hand, the SLR1 protein interacts with the OsGAMYBL2 protein and represses its binding to the target gene promoter. Nevertheless, it was suggested that the OsmiR159d-*OsGAMYBL2* module functions specifically in the BR signaling, but not in the GA signaling pathway. Importantly, the GA signaling promotes degradation of the OsGAMYBL2 protein, and consequently enhances the BR signaling. Generally, these results indicate that molecular mechanism of the BR-GA signaling interplay is highly complicated [117] (Figure 3).

Regulation of the leaf inclination in rice may be also mediated through synergistic interaction between the BR and auxin signaling [100]. The rice LPA1 (Loose Plant Architecture1) protein influences plant architecture and the auxin homeostasis [130,131]. The are two BR-mediated pathways that interact with auxin to regulate the leaf inclination in rice: the BR biosynthesis-dependent pathway and the OsBRI1-mediated pathway [9]. LPA1 represses the auxin signaling that interacts with the C-22-hydroxylated and 6-deoxo BRs, therefore, it regulates the lamina joint bending independently of the OsBRI1-mediated pathway (Figure 3). However, no interaction between the LPA1 and OsBRI1 proteins was reported. The extent of leaf inclination is negatively correlated with the *LPA1* gene expression (the higher *LPA1* gene expression the more erect phenotype). Therefore, LPA1 suppresses the joint lamina bending in rice. Mutant of the *LPA1* gene is hypersensitive to auxin during the leaf inclination which is suppressed by the BR-biosynthesis inhibitor—brassinazole. It was reported that LPA1 stimulates expression of the *OsPIN* genes, which indicates that auxin transport plays a key role in the LPA1-dependent lamina joint bending in rice (Figure 3). Thus, it was concluded that there are two independent pathways regulating the auxin-dependent lamina inclination in rice which proceed either through BR biosynthesis or signaling [131].

The interaction between auxin and BR signaling pathways is also mediated by the OsEMF1 transcription regulator which is involved in the histone methylation-dependent epigenetic gene silencing [57]. In rice OsEMF1 plays a role in palea development through maintaining the epigenetic repression of the *OsMADS58* gene [132,133]. Recently, a mutant of the *OsEMF1* gene, named *ds1*, with decreased plant height, leaf angle and grain size has been identified. The mutant was reported to be insensitive to BR. As a results of the identified mutation, expression of the BR biosynthetic genes (*OsDWARF4* and *OsD2*) was increased, whereas expression of the BR signaling-related genes (*OsBRI1*, *OsBZR1* and *OsBU1*) was repressed. Interestingly, the OsEMF1 transcription regulator directly interacts with the OsARF11 transcription factor to positively co-regulate expression of the *OsBRI1* gene (Figure 3). Thus, the OsEMF1 transcription factor constitutes a node in the crosstalk between the BR and auxin signaling pathways [57].

It is known that ABA antagonizes the stimulating effect of BR on the joint lamina bending. This inhibitory effect of ABA is mediated through targeting the BR biosynthesis (*OsD11*) and signaling (*OsGSK2* and *OsDLT*) genes. Interestingly, the ABA application stimulated expression of the BR biosynthetic genes *OsD2* and *OsDWF4*, but suppressed expression of another BR biosynthetic gene—*OsD11*. It was postulated that the modulation of the *OsD11* gene expression is important for the BR-ABA crosstalk-mediated regulation of the lamina joint bending. Moreover, the BR feedback-mediated inhibition of the *OsBRI1* gene expression was partially counteracted by the ABA application. However, the OsGSK2 kinase (partially) and the OsDLT transcription factor (chiefly) constitute the major nodes in the ABA-BR signaling crosstalk and the regulation of leaf inclination [7].

## 6. Conclusions

Identification and functional characterization of the BR signaling components in monocots is an ongoing process. This review presented the updated view on the current model of the BR signaling in rice and other cereals. Importantly, the results obtained mainly in rice but also in other monocot species allowed for identification of some BR signaling components and their functions which are unique for the monocots. This has significantly broadened the view on the BR signaling pathway which was described in Arabidopsis. Moreover, the functional analysis of genes encoding the components of the BR signaling in monocots led to isolation of mutants with phenotypic traits which are preferable in the modern agricultural practices, including the semi-dwarfism and erect stature. These traits enable the high-density planting, and in some cases also cause the enhanced tolerance to environmental stresses. At least several genes encoding the components of the BR signaling in cereals may be regarded as the potential targets for modulation of the plant architecture with the application of the genetics-based approaches. Therefore, these results and achievements are of significant importance for elucidating evolution and molecular mechanisms of the BR signaling in monocots and other species, but also for the future breeding programs of cereal cultivars with the improved yield potential and enhanced tolerance to environmental stresses. The development of such improved cereal cultivars seems to be particularly important in the face of ongoing global climate change.
